# Detrimental effects of cerium oxide nanoparticles on testis, sperm parameters quality, and in vitro fertilization in mice: An experimental study

**DOI:** 10.18502/ijrm.v19i9.9712

**Published:** 2021-10-10

**Authors:** Elnaz Hosseinalipour, Mojtaba Karimipour, Abbas Ahmadi

**Affiliations:** ^1^Department of Anatomy and Histology, Faculty of Medicine, Urmia University of Medical Sciences, Urmia, Iran.; ^2^Reproductive Health Research Center, Urmia University of Medical Sciences, Urmia, Iran.; ^3^Department of Basic Sciences, Faculty of Veterinary Medicine, Urmia University, Urmia, Iran.

**Keywords:** Cerium oxide, Testis, Sperm, Fertilization, Mice.

## Abstract

**Background:**

Cerium oxide nanoparticles (CeO${}_{2}$ NPs) as an important nanomaterial have a wide range of applications in many fields and human beings' exposure to this nanomaterial is unavoidable. The effects of CeO${}_{2}$ NPs on the male reproductive system are controversial.

**Objective:**

To determine the effects of the administration of CeO${}_{2}$ NPs on the testis tissue, sperm parameters, and in vitro fertilization (IVF) in mice.

**Materials and Methods:**

Twenty-four male mice were divided into three groups (n = 8/each): one control and two experimental groups receiving CeO${}_{2}$ NPs at doses of 50 and 100 mg/kg body weight, respectively, for 35 days. At the end of the experiment, the diameter of seminiferous tubules (SNTs), epithelial height of SNTs, spermiogenesis index in testes, sperm parameters (count, motility, viability, and morphology), sperm chromatin condensation, DNA integrity, and IVF assays were analyzed.

**Results:**

Histological results showed that the tubular diameter, the epithelial height of the SNTs, and the spermiogenesis index were significantly decreased in the experimental groups receiving CeO${}_{2}$ NPs. All sperm parameters in the experimental groups were significantly reduced and, additionally, the percentages of immature sperms and sperms with DNA damage were significantly increased in groups treated with CeO${}_{2}$ NPs compared to the control. Furthermore, the rates of IVF and in vitro embryo development were decreased.

**Conclusion:**

Collectively, the current study showed that oral administration of CeO${}_{2}$ NPs in mice had detrimental effects on the male reproductive system through inducing testicular tissue alterations, decreasing sperm parameters quality, and also diminishing the IVF rate and in vitro embryonic development.

## 1. Introduction

Due to the growth of nanotechnology, studies related to the health risk of nanomaterials have been increasing rapidly (1). Nanoparticles (NPs) are material particles 1-100 nm in size and are commonly used in many industrial products (2). It has been reported that more than 1,814 nanomaterials are now used in different products (3). The size of these materials permits them to pass through biological membranes and barriers and penetrate the cells (4) and ultimately induce adverse effects such as oxidative stress, inflammation, gene mutations, and cell death (5, 6). NPs are widely used in medicine and their therapeutic application has been largely due to their oxidative stress-alleviating ability (7).

Nanoceria or cerium oxide nanoparticles (CeO${}_{2}$ NPs) are oxides of rare earth elements and are widely and extensively used in many industries such as the automotive industry, wood care application, and medicine (8, 9). These NPs are also used as diesel additives to reduce fuel consumption and decrease CO${}_{2}$ gas production (10). Due to the high emission of CeO${}_{2}$ NPs, the Organization for Economic Cooperation and Development has included these NPs in the list of NPs that need to be evaluated with high priority (11).

The existing publications related to the potential adverse effects of CeO${}_{2}$ NPs are largely controversial. Some reports have shown that CeO${}_{2}$ NPs are antioxidant agents that improve the total antioxidant capacity and prevent oxidative stress damage (12-14). CeO${}_{2}$ NPs have also been shown to possess anti-inflammatory properties (15). Conversely, several studies have reported the pro-oxidant effects and DNA damage in somatic cells and mouse oocytes following CeO${}_{2}$ NPs exposure (11, 16).

The publications on the effects of CeO${}_{2}$ NPs on the reproductive system are also controversial. In male mice, exposure to CeO${}_{2}$ NPs can lead to reproductive toxicity (17). In another study, it was shown that exposure of mice oocytes to CeO${}_{2}$ NPs caused oxidative stress and DNA damage (11). On the contrary, several publications have demonstrated the beneficial effects of CeO${}_{2}$ NPs on the reproductive system. For example, in a recent study, it was documented that CeO${}_{2}$ NPs improve oxidative stress-induced testicular toxicity due to cyclophosphamide administration (18). Or in another study, it was exhibited that exposure of ram sperm to CeO${}_{2}$ NPs had beneficial effects on motility and plasma membrane integrity (19). Furthermore, it was reported that pretreatment with CeO${}_{2}$ NPs relieved the adverse effects of radiofrequency radiation on testosterone synthesis through antioxidant capacity, enhanced the expression of testosterone synthase, and also elevated the resistance to oxidative damage in Leydig cells (20).

However, the available information regarding the adverse effects of CeO${}_{2}$ NP administration on the male reproductive system is controversial and incomplete. Thus, the objective of the present study was to determine the effects of in vivo CeO${}_{2}$ NPs exposure in male mice on testicular tissue, sperm parameters, sperm DNA fermentation, in vitro fertilization (IVF) rates, and in vitro embryo development. To the best of our knowledge, no publication has been reported on the effects of in vivo CeO${}_{2}$ NPs exposure in male mice on IVF and in vitro embryo growth.

## 2. Materials and Methods

### Animal and treatment

In this experimental study, 24 adult male NMRI mice (8-10 wk old, 25-27 gr) were kept under standard animal house conditions (23 ± 2°C; 12-hr light/dark cycles, and ad libitum access to food and water). The study was performed in the Anatomy and Histology Department of Urmia University of Medical Sciences, Urmia, Iran, between 2018 and 2019. After a one wk adaptation to the new environment, the animals were randomly divided into one control group and two experimental groups (n = 8/each) that received daily oral administration of CeO${}_{2}$ NPs at doses of 50 or 100 mg/kg body weight, respectively, for 35 consecutive days. The CeO${}_{2}$ NPs used in the current study (30 nm in diameter with 99.97% purity) were obtained from US Research Nanomaterials, Inc, USA. NPs were suspended in normal saline solution and vortexed before every administration. The mice in the control group received normal saline as the vehicle. The duration of the study was selected based on the timing (35 days) of the mouse spermatogenesis process reported in previous studies (21).

At the termination of the study, the mice were euthanized with an intraperitoneal injection of ketamine and testicular histomorphometry (seminiferous tubule diameter and epithelial height), spermiogenesis index (SPI), sperm parameters, and IVF assay were evaluated.

For evaluating the testicular histomorphometry and SPI, the left testes of each mouse was removed and fixed immediately in 10% formalin. Following the tissue processing, the 5-μm thick sections stained with hematoxylin and eosin were obtained. Then, 10 round-shaped tubules from each sample were randomly selected and the diameter of the seminiferous tubules (SNTs) and epithelial height were measured. In each SNT, the average of two diameters perpendicular to each other were determined. At two locations, the thickness from the basement membrane to the luminal surface was measured to obtain the average epithelial thickness of the same SNT (22, 23).

To determine the SPI in each sample, 20 SNTs were randomly selected and the percentage of SNTs with sperm in the lumen was calculated.

### Collection of spermatozoa for the evaluation of sperm parameters

After 35 days, the animals were euthanized with an intraperitoneal injection of ketamine. To obtain sperm, both caudal part of the epididymides of each animal were cut into small pieces in pre-warm dishes containing 1-mL human tubal fluid medium (HTF; Sigma, USA) supplemented with 4 mg/mL bovine serum albumin (BSA; Sigma, USA) and incubated at 37°C in 5% CO${}_{2}$ to release sperm from the epididymis into the medium.

After diluting the samples, sperms were counted using a Neubauer slide under a light microscope with 400× magnification. To assess the sperm motility, 10 μL of sperm sample was placed on a pre-warm Neubauer slide, and then, the percentage of motile sperms was calculated.

An aliquot (20 μL) of eosin solution was mixed with the same volume of sperm suspension to assess the sperm viability. Then 20 μL of nigrosine was added and a smear prepared. The slides was dyed at room temperature and the percentage of unstained colorless alive sperms and red-stained dead sperms was determined. At the end, the aniline blue staining method was implemented to evaluate the sperm morphology and the percentage of sperms with abnormal morphology was determined (23, 24).

### Assessment of sperm DNA damage

Acridine orange is a fluorescence stain that can detect double- and single-stranded regions in sperm chromatin. Briefly, the sperm slides were placed in Carnoy's fixative (methanol/acetic acid 1:3) and after drying at room temperature, the slides were transferred to acridine orange solution. Eventually, using a fluorescent microscope, 200 sperms in each sample were evaluated and the percentage of normal sperms (green color) and abnormal ones (yellow to red color) was determined (23).

### Assessment of sperm chromatin quality

Sperm nucleus maturity was analyzed using aniline blue staining. Briefly, the sperm smear slides were fixed in 3% glutaraldehyde and then stained with 5% aniline blue solution. In this test, abnormal immature sperms become dark and the normal sperms appear pale. At least 200 sperms were counted in each slide and the data were presented as a percentage (23).

### IVF assays

#### Oocytes collection and assessment of fertilization and embryonic development

Sixty adult female NMRI mice (8-10 wk old) were used to obtain enough oocytes for IVF assays. In order to induce superovulation and obtain mature oocytes from oviducts, female mice were intraperitoneally injected with 10 IU of pregnant mare's serum gonadotropin (PMSG; Folligon, Netherlands), followed by an injection of 10 IU of human chorionic gonadotropin (hCG; Folligon, Netherlands) 48 hr later. Next, 12-14 hr after the hCG injection, the mice were sacrificed and the fallopian tubes from each mouse were transferred to a petri dish containing HTF-BSA medium previously equilibrated in an incubator with 5% CO${}_{2}$ at 37°C. After dissecting the oviducts, the oocytes were removed and washed, and then placed in the droplets of HTF-BSA medium which were under mineral oil. In order to evaluate IVF, 1 × 10${}^{6}$ capacitated sperms from each sample were added to the oocytes. The fertilization process was evaluated under an inverted microscope after 3-5 hr by observing two pronuclei. Then these zygotes were cultured for 24 hr and the number of two-cell embryos was counted. At the end, the percentages of blastocysts formation after 120 hr and arrested embryos were determined (23, 25, 26).

### Ethical considerations

The study was approved by the Ethics Committee of Urmia University of Medical Sciences and was performed according to the Guidelines for Care and Use of Laboratory Animals (Code: IR.umsu.rec.1395.228).

### Statistical analysis

Statistical analysis for the data of testicular histomorphometry, SPI, and sperm parameters were done using SPSS 16. A one-way ANOVA with Tukey's post-hoc test was performed to quantify the results. The data of IVF assay were analyzed by two proportional test using Minitab software (version 15.1, Minitab Inc, USA). All results were expressed as means ± SD and p-values < 0.05 were considered statistically significant.

## 3. Results

### Effects of CeO${}_{2}$ NP exposure on SPI and testicular histomorphometry

Microscopic analysis showed a significant reduction in the percentage of SNTs with positive SPI in the groups treated with 50 and 100 mg/kg CeO${}_{2}$ NPs compared with the control group (p = 0.005 and p = 0.002, respectively). Moreover, the mice in the CeO${}_{2}$ NPs receiving groups exhibited a significant decrease in the SNTs diameter (p = 0.003 and p < 0.001, respectively) and epithelial height (p = 0.047 and p = 0.007, respectively) in comparison with the control mice (Table I).

### Effects of CeO${}_{2}$ NPs exposure on sperm parameters

Sperm parameters were evaluated 35 days after the NP administration. Sperm count in groups exposed to 50 and 100 mg/kg of CeO${}_{2}$ NPs was significantly reduced (p = 0.003 and p = 0.007, respectively) compared to the control group (Table I). Sperm motility in groups receiving 50 and 100 mg/kg was also significantly decreased compared to the control (p = 0.026 and p < 0.001, respectively). Moreover, it was found a significant decrease (p < 0.001) in the percentage of sperm viability with a dose of 100 mg/kg (Table I). As illustrated in Table I, the percentage of sperm with normal shape in mice exposed to CeO${}_{2}$ NPs was significantly decreased (p < 0.001) compared to the control group.

### Effects of CeO${}_{2}$ NPs exposure on sperm chromatin condensation and DNA integrity

**Figure 1:**
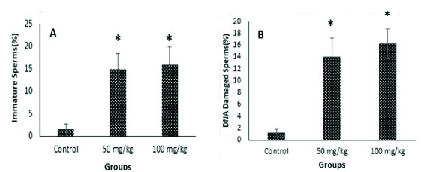
Effects of CeO${}_{2}$ NPs on sperm chromatin condensation and DNA integrity in mice for 35 consecutive days. Values are presented as Means ± SD. Significant difference: CeO${}_{2}$ NPs groups compared with the control group, *P < 0.001.

Figure 1 shows aniline blue and acridine orange staining tests and these results indicated that the percentages of immature sperms and DNA damage in sperms in groups exposed to CeO${}_{2}$ NPs were significantly higher (p < 0.001) than in the control group.

### Effects of CeO${}_{2}$ NPs exposure on IVF rate and development of embryos

**Figure 2:**
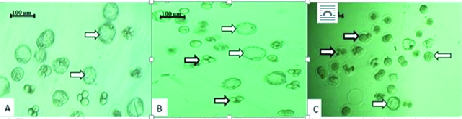
Photomicrograph from pre-implantation embryos. (A) Control group: Normal embryos which most of them are at the blastocyst stage (thin arrows). (B) 50 mg/kg of CeO${}_{2}$ NPs group: Some of the embryos are arrested and lysed (thick arrows). (C) 100 mg/kg of CeO${}_{2}$ NPs group: Most of the embryos are arrested and lysed (inverted microscope, 400×).

As shown in Table II, the percentages of fertilization and two-cell embryos formation in the group receiving 100 mg/kg CeO${}_{2}$ NPs were significantly lower (p < 0.01 and p < 0.001 respectively) than in the control, while no significant difference was observed between the group exposed to 50 mg/kg of CeO${}_{2}$ NPs and the control groups (p = 0.873 and p = 0.429, respectively). Additionally, the percentage of blastocyst formation was also reduced in both of the experimental groups compared to the control group (p < 0.001). Furthermore, the total percentage of arrested embryos was significantly higher (p < 0.001) in the groups receiving CeO${}_{2}$ NPs compared to the control group (Figure 2).

**Table 1:** The effects of oral administration of CeO${}_{2}$ NPs on different parameters between the groups


**Groups**	**Spermiogenesis index (%)**	**Tubular diameter (μm)**	**Epithelial height (μm)**	**Sperm count (×10${}^{6}$ /ml)**	**Sperm motility (%)**	**Sperm viability (%)**	**Normal morphology (%)**
**Control**	86.83 ± 5.11	193.66 ± 4.84	80.16 ± 7.93	35.75 ± 2.46	67.75 ± 5.31	73.25 ± 4.78	87 ± 4.69
**50 mg/kg**	68.83 ± 9.86${}^{b}$	178.83 ± 8.01${}^{b}$	65.33 ± 8.95${}^{a}$	22 ± 4.41${}^{b}$	54.75 ± 6.65${}^{a}$	62 ± 7.07	58.5 ± 5.44${}^{c}$
**100 mg/kg**	66.32 ± 9.09${}^{b}$	174.33 ± 5.75${}^{c}$	59.83 ± 11.95${}^{b}$	23.37± 5.54${}^{b}$	47.5 ± 5.19${}^{c}$	52.5 ± 2.64${}^{c}$	53.5 ± 7.14${}^{c}$
All data are presented as Means ± SD, analyzed by one-way ANOVA test.${}^{a,}$ ${}^{b,}$ ${}^{c}$ Significant difference vs. control (p < 0.05, p < 0.01, and p < 0.001, respectively)							

**Table 2:** The effects of oral administration of CeO${}_{2}$ NPs on in vitro fertility and embryonic development in different groups


**Groups**	**Number of oocytes**	**Fertilization rate**	**Two cell embryos**	**Blastocyst**	**Arrested embryos**
**Control**	187	180 (96.26)	154 (85.55)	111 (61.66)	69 (38.33)
**50 mg/kg**	172	165 (95.93)	136 (82.42)	44 (26.67)${}^{b}$	121 (73.33)${}^{b}$
**100 mg/kg**	338	281 (83.14)${}^{a}$	163 (58)${}^{b}$	42 (14.94)${}^{b}$	239 (85.05)${}^{b}$
Data are presented as number (%). Analyzed by 2 proportional test,${}^{a,b}$ Significant difference vs. control (p < 0.01 and p < 0.001 respectively)					

## 4. Discussion

The existing data from various experiments on potential toxic effects of cerium oxide on the male reproductive system are controversial. In the current study, we used sperm parameters and in vitro fertility potential evaluations to determine the toxic effects of CeO${}_{2}$ NPs on the male reproductive function. The findings of the current study revealed that all sperm parameters (count, motility, maturity, and morphology) had a significant decrease in mice following the CeO${}_{2}$ NPs exposure. Our findings also demonstrated that CeO${}_{2}$ NPs exposure induced adverse effects on IVF rate and embryonic development.

Sperm motility is known as a predictor of male fertility potential because it reflects the ability of sperm to fertilize the oocyte in the female genital tract (27). However, impairment of sperm motility leads to a decrease in the fertilization rate in both in vitro and in vivo.

Several experimental studies have previously reported conflicting findings regarding CeO${}_{2}$ NPs exposure (11, 14, 17, 18, 28-30). In the present study, sperm count and motility were significantly decreased following 35 consecutive days of CeO${}_{2}$ NPs administration. In the current study, the reduction in sperm count was also confirmed by evaluating SPI: it was observed that the percentage of SNTs without sperm in the lumen was significantly decreased in groups receiving CeO${}_{2}$ NPs. This is in agreement with a previous study that found that oral administration of CeO${}_{2}$ NPs led to a significant decrease in sperm motility and daily sperm production (17). A decline in sperm motility was also reported in mice whose mothers were exposed to a high dose of CeO${}_{2}$ NPs during pregnancy, and the testicular tissue examination in these mice showed that the number of spermatogonia, Sertoli, and Leydig cells was significantly decreased (28). In line with our study, Perrin and colleagues showed that in vitro exposure of human sperm cells to CeO${}_{2}$ NPs induced damage due to the aggregation of NPs in the membrane of sperm (29). Conversely, in vitro exposure with CeO${}_{2}$ NPs in rams did not affect sperm motility or the other sperm parameters which are known as indicators of fertility (30). In another study, it was also indicated that CeO${}_{2}$ NPs administration ameliorates male rats' age-related infertility by elevating sex hormones production, and sperm concentration, as well as activating the spermatogenesis process through the antioxidant property (31).

The decrease in the quality of sperm parameters following CeO${}_{2}$ NPs administration may be induced due to translocation of CeO${}_{2}$ NPs from blood circulation to testicular tissue and damage to germ cells or directly induced damage to mature sperms. In a previous study, it was indicated that CeO${}_{2}$ NPs were found in the testes and epididymis of rats following inhalation (32).

Sperm morphology is another parameter that was evaluated in the current study and the results showed that both of the doses of CeO${}_{2}$ NPs (50 and 100 mg/kg) significantly increased the percentages of sperms with an abnormality in morphology. This parameter can also reveal the adverse effects of the chemical on the spermatogenesis process.

It has been indicated that spermatozoa with abnormal morphology is directly related to male infertility and can decline fertilization and pregnancy rates. In the literature, several mechanisms for the cause of abnormality in sperm morphology have been presented, including abnormal chromosome, point mutation in testicular DNA, and also impairment in the differentiation of spermatozoa during spermatogenesis following toxicant exposure (24, 33).

Regarding the sperm viability, our findings indicated that the oral administration of CeO${}_{2}$ NPs significantly decreased the percentages of alive sperms compared to the control group. This finding may have been caused by the direct effect of CeO${}_{2}$ NPs on the testis. However, further studies are needed to clarify this. Contrary to the results of our study, in a previous report, it was shown that exposure to CeO${}_{2}$ NPs for 14 days in rats decreases sperm and testicular damage induced by diabetes (34).

In the current study, we used aniline blue staining to evaluate the nucleus maturity and chromatin defects in sperm. The results showed that CeO${}_{2}$ NPs exposure via oral administration increased chromatin abnormality in sperm. Considering sperm maturation occurs in the epididymis, one possibility is that CeO${}_{2}$ NPs also induce their toxic effects in the epididymis. This result may be associated with oxidative stress damage induced by CeO${}_{2}$ NPs, although the potential of CeO${}_{2}$ NPs to induce oxidative stress is controversial.

The acridine orange staining test showed that CeO${}_{2}$ NPs caused DNA damage-DNA disintegration-in spermatozoa. This damage may transfer the abnormal genome into the oocyte which could reduce the fertilization and pregnancy rates. The IVF assay in the current study revealed that the percentages of fertilization and in vitro embryonic growth in mice exposed to CeO${}_{2}$ NPs were significantly decreased. There are conflicting reports about the genotoxic effects of CeO${}_{2}$ NPs. In a report by Preaubert and colleagues (35), it was shown that exposure to CeO${}_{2}$ NPs causes a significant increase in the DNA damage in mice sperm. Another study also confirmed the genotoxic effects of this NP in human spermatozoa (30), but conversely, no genotoxic effect was reported in ram spermatozoa exposed to CeO${}_{2}$ NPs (19). These aforementioned discrepancies indicate that the effects of CeO${}_{2}$ NPs on sperm may depend on several factors such as species sensitivity, surface chemistry, structure, size, and exposure time and dose.

Histomorphometric analyses in the testicular tissue showed significant decrease in epithelial height and diameter of SNTs in mice receiving CeO${}_{2}$ NPs comparing the control group. These alterations could be related to the decrease in the number of Sertoli cells because there is a direct correlation between the number of Sertoli cells and epithelial height and diameter of SNTs (36, 37). In the present study, the number of Sertoli cells was not evaluated. Further studies are needed to elucidate this relationship.

In addition to evaluating the testicular tissue and sperm parameters in the current study, we also evaluated the IVF assay. To the best of our knowledge, the current study is the first report describing the effects of in vivo exposure to CeO${}_{2}$ NPs on in vitro fertility and embryonic development. Findings from the present study indicated that oral administration of CeO${}_{2}$ NPs in male mice led to a significant decrease in the rate of fertilization and the percentages of two-cell embryos and blastocyst formation. The percentage of arrested embryos also increased. Sperm parameters quality is directly correlated to fertility and embryonic growth. The exact mechanisms by which CeO${}_{2}$ NPs caused damage to sperm, fertility, and embryonic growth were not obtained from the present study. Further investigation is required to support these findings and explore the involved mechanisms.

Our study had some limitations. For example, evaluations of oxidative stress and apoptosis in testicular tissue following CeO${}_{2}$ NPs exposure can provide additional information about mechanisms involved in CeO${}_{2}$ NPs toxicity.

## 5. Conclusion

In the current study, long-term oral administration of CeO${}_{2}$ NPs at doses of 50 and 100 mg/kg impaired male reproductive functions by decreasing sperm parameters quality and inducing sperm DNA damage and testicular tissue alterations in mice. In addition, it was also observed that male mice receiving CeO${}_{2}$ NPs showed a decreased IVF rate and in vitro embryonic development. These findings provide useful information on the risk of CeO${}_{2}$ NPs application on the function of the male reproductive system. Finally, further investigations are needed to examine the potential influence of these particles on spermatogenesis in men.

##  Conflict of Interest

The authors report no conflict of interest.
